# Genome Sequences of Five Disinfectant-Resistant Listeria monocytogenes Strains from Two Iberian Pork-Processing Plants

**DOI:** 10.1128/genomeA.00077-15

**Published:** 2015-03-05

**Authors:** Victoria López-Alonso, Sagrario Ortiz, Joaquín V. Martínez-Suárez

**Affiliations:** a Unidad de Biología Computacional, UFIEC, Instituto de Salud Carlos III, Majadahonda, Madrid, Spain; b Departamento de Tecnología de Alimentos, Instituto Nacional de Investigación y Tecnología Agraria y Alimentaria (INIA), Madrid, Spain

## Abstract

We announce the draft genome sequences of five Listeria monocytogenes strains from two Iberian pork-processing plants located in Spain that were distinguished by their resistance to benzalkonium chloride. These strains seem highly adapted to the meat-processing environment according to their persistence and transmission capabilities.

## GENOME ANNOUNCEMENT

Listeria monocytogenes is an important foodborne pathogen with an outstanding capacity to persist in the food-processing plants (1). The persistence of specific L. monocytogenes strains after industrial cleaning and disinfection suggests that resistance to disinfectants, such as benzalkonium chloride (BAC), may be related to the mechanisms employed by L. monocytogenes for environmental survival, colonization of the processing plant, and transmission.

We have previously shown that control of L. monocytogenes contamination in an Iberian pig abattoir and processing plant (plant A) originated the selection of BAC-resistant strains of certain persistent pulsed-field gel electrophoresis (PFGE) types such as S1 and S10-1 (2). The study of a geographically separated ready-to-eat meat-processing plant of the same company (plant B) revealed the presence of subtypes S1 and S10-1 together with three additional BAC-resistant PFGE types denominated S2-2, S2-3, and S10-3 (S. Ortiz, V. López, P. Rodríguez, and J. V. Martínez-Suárez, unpublished data). Here, one isolate for each PFGE type was subjected to whole-genome shotgun sequencing. Isolates are indicated as PFGE type plus isolation date [ddmmyy]. Two isolates were obtained in 2008 in plant A (S1 [160908] and S10-1 [160908]), and three were obtained in 2010 in plant B (S2-2 [260510], S2-3 [240810], and S10-3 [161210]).

All L. monocytogenes strains where grown in tryptic soy yeast extract broth (Biolife, Milan, Italy) at 37°C. Genomic DNA was extracted using a bacterial genomic DNA purification kit (Wizard, Promega, Madison, WI, USA) according to the manufacturer’s protocol. The library was prepared from the extracted genomic DNA using TruSeq technology (Illumina, San Diego, CA, USA) and a 2× 250-nucleotide paired-end sequencing run was performed in a MiSeq platform. The reads were trimmed and then assembled *de novo* into a high-quality draft genome using the Spades 3.1.1 software (3). The genomes were assembled to 2,997,617 bp with 282-fold sequencing coverage for S1, 3,009,749 bp with 219-fold sequencing coverage for S10-1, 3,086,604 bp with 259-fold sequencing coverage for S2-2, 3,093,105 bp with 314-fold sequencing coverage for S2-3, and 3,1114,032 bp with 254-fold sequencing coverage for S10-3. The genomes were annotated with the NCBI Prokaryotic Genomes Automatic Annotation Pipeline (PGAAP) (4) and the Rapid Annotation using Subsystem Technology (RAST) server (5). The number of predicted genes ranges from 3,036 to 3,193 (average, 3,114), including RNAs (average, 73). A preliminary analysis located numerous genes coding transport proteins which could be involved in disinfectant resistance (S. Ortiz, V. López, P. Rodríguez, and J. V. Martínez-Suárez, unpublished data).

The availability of these L. monocytogenes genome sequences will facilitate the study of the mechanism that contributes to the environmental persistence of L. monocytogenes upon contact with disinfectants.

### Nucleotide sequence accession numbers.

This whole-genome shotgun project has been deposited at DDBJ/EMBL/GenBank under the accession numbers JWHI00000000, JWHG00000000, JWHJ00000000, JWHK00000000, and JWHH00000000, for isolates S1 [160908], S10-1 [160908], S2-2 [260510], S2-3 [240810], and S10-3 [161210], respectively.
